# Hair graying with aging in mice carrying oncogenic *RET*

**DOI:** 10.1111/acel.13273

**Published:** 2020-11-07

**Authors:** Machiko Iida, Akira Tazaki, Ichiro Yajima, Nobutaka Ohgami, Nobuhiko Taguchi, Yuji Goto, Mayuko Y. Kumasaka, Armelle Prévost‐Blondel, Michihiro Kono, Masashi Akiyama, Masahide Takahashi, Masashi Kato

**Affiliations:** ^1^ Department of Occupational and Environmental Health Nagoya University Graduate School of Medicine Nagoya Japan; ^2^ Unit of Environmental Health Sciences Department of Biomedical Sciences College of Life and Health Sciences Chubu University Kasugai‐shi Japan; ^3^ General Research and Development Institute Hoyu Co Ltd Nagakute‐shi Japan; ^4^ INSERM U1016 CNRS UMR8104 Université de Paris Cochin Institute Paris France; ^5^ Departments of Dermatology Nagoya University Graduate School of Medicine Nagoya Japan; ^6^ Departments of Molecular Pathology Nagoya University Graduate School of Medicine Nagoya Japan; ^7^ Present address: Department of Bioscience and Engineering College of Systems Engineering and Science Shibaura Institute of Technology Minuma‐ku Japan; ^8^ Present address: Department of Dermatology and Plastic Surgery Akita University Graduate School of Medicine Akita Japan; ^9^ Present address: International Center for Cell and Gene Therapy Fujita Health University Toyoake Japan

**Keywords:** age‐related hair graying, endothelin, keratinocyte stem cells, melanocyte stem cells, RET

## Abstract

Hair graying is a representative sign of aging in animals and humans. However, the mechanism for hair graying with aging remains largely unknown. In this study, we found that the microscopic appearance of hair follicles without melanocyte stem cells (MSCs) and descendant melanocytes as well as macroscopic appearances of hair graying in *RET*‐transgenic mice carrying *RET* oncogene (RET‐mice) are in accordance with previously reported results for hair graying in humans. Therefore, RET‐mice could be a novel model mouse line for age‐related hair graying. We further showed hair graying with aging in RET‐mice associated with RET‐mediated acceleration of hair cycles, increase of senescent follicular keratinocyte stem cells (KSCs), and decreased expression levels of endothelin‐1 (ET‐1) in bulges, decreased endothelin receptor B (Ednrb) expression in MSCs, resulting in a decreased number of follicular MSCs. We then showed that hair graying in RET‐mice was accelerated by congenitally decreased Ednrb expression in MSCs in heterozygously Ednrb‐deleted RET‐mice [Ednrb(+/−);RET‐mice]. We finally partially confirmed common mechanisms of hair graying with aging in mice and humans. Taken together, our results suggest that age‐related dysfunction between ET‐1 in follicular KSCs and endothelin receptor B (Ednrb) in follicular MSCs via cumulative hair cycles is correlated with hair graying with aging.

## Graphical abstract

We have generated a model mouse for age‐related hair graying. Age‐related hair graying proceeds by increased senescence of KSCs by cumulative hair cycles. This leads to decreased expression of ET‐1 in KSCs, followed by decreased expression of Ednrb in MSCs, resulting in depletion of MSCs and descendant melanocytes.

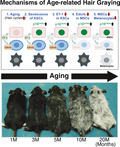

## 1 INTRODUCTION

Hair graying is one of the typical signs of aging in animals and humans. Previous studies have suggested that a decrease in the number of follicular melanocyte stem cells (MSCs) with aging is one of the fundamental causes of hair graying in humans (Nishimura et al., 2005). Decades are needed to analyze the dynamics of hair graying with aging in humans. Therefore, model animals that progressively develop hair graying with aging, which was defined as age‐related hair graying in this study, are useful tools for analyzing the mechanisms of hair graying. To our knowledge, more than 10 kinds of genetically modified mice with hair graying have been reported (Kurita et al., 2005; Moriyama et al., 2006; Tanimura et al., 2011), while wild‐type mice with hair graying are very limited (Endou et al., 2014; Inoue‐Narita et al., 2008; Nishimura et al., 2005). However, analysis of age‐related hair graying is difficult even in the model mice because many of the animals are models for premature hair graying with a limited life span rather than age‐related hair graying. If model animals with a lifespan of >20 months are available, it may be possible not only to obtain new insights into age‐related hair graying but also to develop novel anti‐hair graying medicines.

The hair cycle consists of the anagen phase (growth phase), catagen phase (regression phase), and telogen phase (resting phase) (Müller‐Röver et al., 2001). Since division and self‐renewing of follicular keratinocyte stem cells (KSCs) and MSCs are observed in every hair cycle (Cotsarelis, 2006; Nishimura et al., 2002), increased hair cycles may necessarily involve accumulation of cell division and self‐renewing of follicular KSCs and MSCs. Therefore, we assume that increased hair cycles may promote hair graying via senescence of KSCs and/or MSCs. Senescence markers including p16^ink4a^ and senescence‐associated beta‐galactosidase (SA‐ß‐gal) have been established in previous studies (Baker et al., 2011; Ressler et al., 2006). Correspondingly, hair graying is also promoted through depilation (Endou et al., 2014; Inoue‐Narita et al., 2008). However, hair graying might be caused by factors other than repeated hair cycles such as apoptosis of bulge cells (Ito et al., 2002) and skin inflammation (Chen et al., 2015). Thus, an experiment on hair depilation may not be a suitable experiment for understanding age‐related hair graying.

Recent studies on hair graying in mice have shown that a microenvironment of MSCs, known as a niche of MSCs, is formed by KSCs and plays a crucial role in survival of MSCs. Notch (Moriyama et al., 2006), stem cell factor (SCF) (Endou et al., 2014), and transforming growth factor beta (TGF‐ß) signals (Nishimura et al., 2010) from follicular KSCs have been reported to be essential for the development and maintenance of follicular MSCs. Interaction between endothelins (ETs) in follicular KSCs and endothelin receptor B (Ednrb) in follicular MSCs has also been reported to promote proliferation, expansion, and differentiation of MSCs in young mice stably expressing ß‐catenin in keratin 15‐positive keratinocytes (Rabbani et al., 2011; Takeo et al., 2016). To our knowledge, however, there have been very few studies showing the effects of age‐related dysfunction of follicular KSCs and MSCs on hair graying in animals.

c‐RET proto‐oncogene product, a receptor type of protein tyrosine kinase, is expressed at the inner and outer root sheaths of hair follicles in mice and humans and has been suggested to be associated with regulation of hair cycles (Kato et al., 2001). Activity of c‐RET is strongly correlated with phosphorylation level of tyrosine 905 (Y905) of c‐RET (Kato et al., 2002). A RET‐transgenic mouse line was established by a construct carrying human constitutively activated RET (*RFP*‐*RET*), driven by mouse *metallothionein*‐*1* (MT1) promoter–enhancer (Kato et al., 1998, 1999, 2004). MT1 is expressed in the hair matrix of the bulb, in the outer root sheath of the hair follicle, and in basal cells of the interfollicular epidermis but not in the dermal papillae in normal skin in the back of mice (Karasawa et al., 1991). Our previous study showed spontaneous development of skin melanomas in RET‐mice (Kato et al., 1998). Since previous studies showed the coincidence of hair graying and melanoma from identical genetic mutations in horses and patients with Werner syndrome, respectively (Goto, 2000; Rosengren Pielberg et al., 2008), coincidence of melanoma and hair graying in RET‐mice is possible. Although hair graying in RET‐mice was already observed when the first generation of RET‐mice with a C57BL/6 background was produced (Kato et al., 1998), the phenotype has not been reported.

In this study, we established a model mouse line carrying *RFP*‐*RET* for hair graying with aging. We then investigated the mechanisms of hair graying with focus on age‐related degradation of the interaction between follicular KSCs and MSCs. We finally investigated the similarity in mechanisms of age‐related hair graying in our model mice and humans.

## 2 RESULTS

### 2.1 Hair graying in RET‐mice

**Figure 1:**
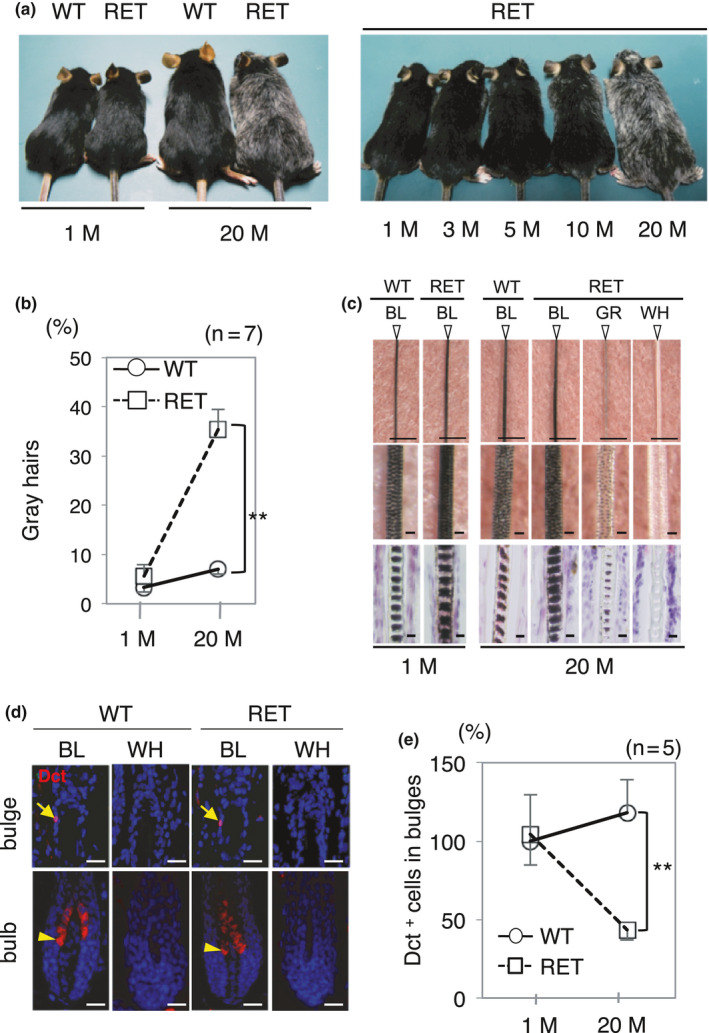
Age‐related hair graying with reduction of MSCs in RET‐mice. (a) Representative macroscopic appearances of WT‐mice and RET‐mice at 1 and 20 months of age (left) and RET‐mice at indicated ages (right). (b) Ratios (means ± SD) of gray hairs (100 hairs being counted for each mice) in WT‐mice (n = 7) and RET‐mice (*n* = 7) at 1 and 20 months of age. (c) Representative photographs of black (BL), gray (GR), and white (WH) hair from WT and RET‐mice at 1 month of age and 20 months of age. Bars, 0.5 mm (Top), 10 µm (middle and bottom). (d) Representative expression of Dct (red) in bulges (top panels, arrows) and in bulbs (bottom panels, arrowheads) in black (BL) and white (WH) anagen follicles from WT‐mice and RET‐mice at 20 months of age. Nuclei were stained with DAPI (blue). Bars, 10 µm. (e) Ratios (means ± SD) of total number of Dct‐positive cells (MSCs) in 70 bulges from WT‐mice at 20 months of age (*n* = 5) and RET‐mice at 1 (*n* = 5) and 20 months of age (*n* = 5) to that in 70 bulges from WT‐mice at 1 month of age (*n* = 5) are presented. **, Significantly different (**p < 0.01) by the Mann–Whitney *U* test. M, months

We first noticed that RET‐mice progressively develop hair graying with aging (Figure 1a). More than 35% macroscopically distinguishable gray hairs had developed in 20‐month‐old RET‐mice, while the percentages of gray hairs in 1‐month‐old WT‐mice and littermate RET‐mice were comparable (Figure 1a,b). Although the percentage of gray hairs in 20‐month‐old WT‐mice was also increased compared to that in 1‐month‐old WT‐mice, gray hairs in 20‐month‐old WT‐mice were less than 7% of total hairs and were macroscopically undetectable (Figure 1a,b). In RET‐mice, graying of hairs was generally observed over the whole body (Figure 1a).

We next microscopically examined the mechanism of hair graying with focus on follicular melanocytes. Black hairs from RET‐mice seemed to contain a larger amount of melanin than that in black hairs from WT‐mice (Figure 1c), being in accordance with our previous report (Kato et al., 2001). Dopachrome tautomerase (Dct)‐positive cells in bulges and in bulbs were judged to be follicular MSCs and descendant melanocytes, respectively (Nishimura et al., 2002). Our immunohistochemical analysis using serial sections showed that the mean number (±SD) of MSCs in single telogen bulges from 1‐month‐old WT‐mice was 1.7 ± 0.66. Numbers of MSCs were not significantly different in 1‐month‐old WT‐mice and littermate RET‐mice (Figure 1d,e), whereas the ratio of MSCs in 20‐month‐old RET‐mice was 63.2% lower than that in littermate WT‐mice (Figure 1e). Our analysis using serial sections further showed that MSCs and descendant melanocytes in white hair follicles from 20‐month‐old RET‐mice were undetectable (Figure 1d).

### 2.2 Ret expression in KSCs and acceleration of hair cycles in RET‐mice

Since loss of MSCs with aging is associated with hair graying in RET‐mice, we next examined Ret expression in MSCs. The anti‐RET antibody detects both endogenous Ret (c‐Ret) and transgenic Ret (RFP‐RET). Therefore, Ret expression in WT‐mice shows endogenous c‐Ret protein, while Ret expression in RET‐mice shows overall expression levels of endogenous c‐Ret and introduced constitutively activated RET protein. In our immunohistochemical analysis, c‐Ret/pRET expression was undetectable in Dct‐positive cells (*n* > 30) in telogen, anagen, and catagen bulges from WT‐mice and RET‐mice (Figure S1).

**Figure 2:**
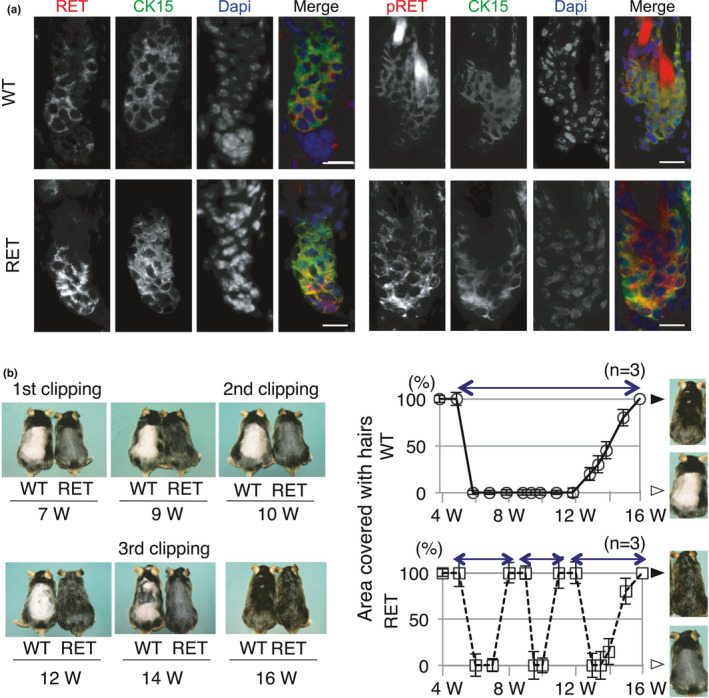
Accelerated hair cycles in RET‐mice. (a) Representative results for expression of c‐Ret/RET (RET; red), cytokeratin 15 (CK15; green), and c‐Ret/RET protein phosphorylated at tyrosine 905 (pRet; red) in telogen bulges at 3 weeks of age are presented. Yellow merged with red and green indicates double staining of CK15 and c‐Ret/RET or phosphorylated c‐Ret/RET proteins in KSCs. Bars, 10 μm. (b) Representative photographs showing macroscopic appearances of hair cycles in WT‐mice (WT, open circle) and RET‐mice (RET, open square) after clipping. Telogen hairs were gently clipped at 7 weeks of age (1st clipping) in both WT‐mice and RET‐mice. Second and 3rd clippings were performed only in RET‐mice at 10 weeks and 14 weeks of age after hairs were fully regenerated. Graphs show ratios (means ± SD) of the skin area covered with regenerated hairs in WT‐mice (*n* = 5) and RET‐mice (*n* = 5) from 4 weeks to 16 weeks of age. Two‐way arrows indicate the length of one hair cycle. Black and white arrowheads beside the graphs show representative photographs of back skin covered and not covered with regenerated hairs. W, weeks

Expression of cRet/pRET was observed in CK15‐positive cells (KSCs) in telogen bulges from both WT‐mice and cRet‐mice (Figure 2a). Expression of cRet/pRET was also observed in KSCs in anagen bulges but not in KSCs in catagen bulges from WT‐mice (Figure S2). In contrast, cRet/pRET expression was detected in KSCs in catagen bulges from RET‐mice (Figure S2). Thus, our results suggest irregular expression of the RET transgene in the catagen phase.

We next examined the effect of constitutively activated RET kinase on the hair cycle. Clipped hairs of RET‐mice were regenerated 3 times during the period of one regeneration cycle in WT‐mice (Figure 2b). Our histological analysis showed that the period of telogen in WT‐mice was from 7 to 12 weeks of age (Figure S3), which correlated well with the results of a previous study (Müller‐Röver et al., 2001). On the other hand, the period of telogen in RET‐mice was found to be less than 1 week (Figure 2b and Figure S3), indicating that the period of telogen in RET‐mice was less than 1/5 of that in WT‐mice. These data suggested that ectopically activated RET kinase in the catagen phase might have caused the shortening of the hair cycle in RET‐mice.

### 2.3 Acceleration of senescence of KSCs in RET‐mice

**Figure 3:**
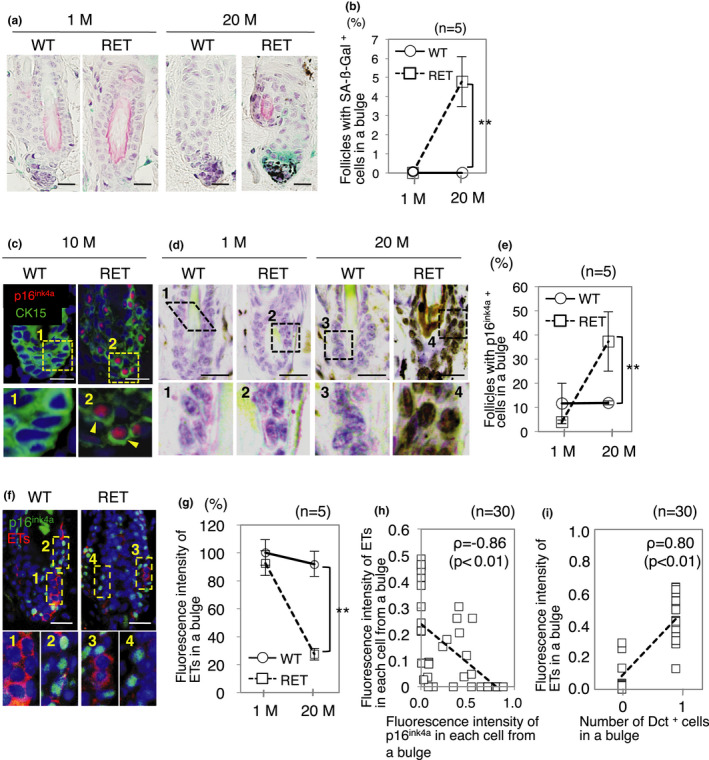
Accelerated senescence of KSCs in RET‐mice. (a, b) Senescence‐associated beta‐galactosidase (SA‐ß‐gal) activities in telogen bulges from WT‐mice and RET‐mice at 1 month of age (1 M) and 20 months of age (20 M) are presented. (a) Photographs of representative SA‐ß‐gal activities (blue) in telogen bulges from WT‐mice and RET‐mice. Nuclei were stained with hematoxylin (purple). (b) Ratios (means ± SD) of follicles with SA‐ß‐gal‐positive cells in telogen bulges (60 each) from WT‐mice (*n* = 5) and RET‐mice (*n* = 5). (c) Representative results for expression of p16^ink4a^ (red) and CK15 (green) in telogen bulges from WT‐ and RET‐mice at 10 months of age. Bottom panels (1, 2) show magnified images of boxed areas in top panels. Expression of p16^ink4a^ protein in the nuclei was detected in CK15‐positive cells (arrowheads) in telogen bulges from RET‐mice. (d) Representative results for expression of p16^ink4a^ in telogen bulges from WT‐ and RET‐mice at 1 and 20 months of age. Bottom panels (1‐4) show magnified images of the boxed areas in the top panels. (e) Ratios (means ± SD) of follicles with p16^ink4a^‐positive cells in telogen bulges (50 each) from WT‐ (*n* = 5) and RET‐mice (*n* = 5) at 1 and 20 months of age. (f) Representative results for expression of p16^ink4a^ (green) and ETs (red) in telogen bulges from WT‐ and RET‐mice at 20 months of age. Bottom panels (1‐4) show magnified images of the boxed areas in the top panels. (g) Ratios (means ± SD) of fluorescence intensity of ETs in telogen bulges (30 each) from WT‐mice at 20 months of age (*n* = 5) and RET‐mice at 1 month of age (*n* = 5) and 20 months of age (*n* = 5) to that in telogen bulges from WT‐mice at 1 month of age (*n* = 5). (h) Correlation between fluorescence intensities of ETs and p16^ink4a^ monitored in the same cell of 30 individual cells in telogen bulges from RET‐mice at 20 months of age. (i) Correlation between fluorescence intensity of ETs and number of Dct‐positive cells detected in the same bulge region (30 follicles) from single tissue sections of 20‐month‐old RET‐mice. **Significantly different (**p < 0.01) by the Mann–Whitney *U* test. Bars, 10 μm. M, months

Lineage tracing studies demonstrated that the bulge acts as a stem cell pool of the hair follicle. These cells are quiescent in general, but division and self‐renewing of KSCs and MSCs are repeated in every hair cycle to replenish the stem cell pool (Cotsarelis, 2006; Nishimura et al., 2002). Therefore, we assumed that age‐related and RET‐mediated increase in hair cycles promotes accelerated senescence of KSCs and MSCs in RET‐mice. Cellular senescence was therefore examined by using a well‐established senescence biomarker, SA‐ß‐gal. We found follicles with cells cytoplasmically stained with SA‐ß‐gal (SA‐ß‐gal‐positive cells) in 20‐month‐old RET‐mice but not in 1‐month‐old and 20‐month‐old WT‐mice or 1‐month‐old RET‐mice were negative (Figure 3a). We further quantified the numbers of follicles with SA‐ß‐gal‐positive cells and found that there was a statistically significant increases in such follicles in 20‐month‐old RET‐mice (Figure 3b). We next examined which cell type, MSCs or KSCs, shows cellular senescence by using another well‐established biomarker, p16^ink4a^ (Baker et al., 2011; Ressler et al., 2006). However, p16^ink4a^ signals were undetectable in Dct‐positive cells in bulges from both WT‐mice and RET‐mice (Figure S4). On the other hand, all of the nuclear stained p16^ink4a^‐positive cells were CK15‐positive cells (KSCs) in bulges from 10‐month‐old WT‐mice and RET‐mice (Figure 3c). Ratios of follicles with p16^ink4a^‐positive cells in bulges from 1‐month‐old WT‐mice, 20‐month‐old WT‐mice, and 1‐month‐old RET‐mice were comparable (Figure 3d,e). However, the ratio of follicles with p16^ink4a^‐positive cells in bulges from 20‐month‐old RET‐mice was significantly higher than the ratios in bulges from littermate WT‐mice and 1‐month‐old RET‐mice (Figure 3d,e).

We also examined the expression of p16^ink4a^ in interfollicular epidermal stem cells, which continuously divide throughout animal life. At one month of age, there were a few p16^ink4a^‐positive cells in the basal epidermis (Figure S5a). There was a statistically significant increase in the number of p16^ink4a^‐positive cells at 20 months of age in both WT‐mice and RET‐mice (Figure S5a,b). These data suggested that the increased number of cell divisions might have led to the up‐regulation of p16^ink4a^.

Since RET is a well‐characterized proto‐oncogene, we further characterized this accelerated senescence phenotype with markers for oncogene‐induced senescence (OIS). Phosphorylation of the histone variant H2AX, forming γH2AX, is an early cellular response to the induction of DNA double‐strand breaks. Formation of γH2AX has been shown to be induced by strong oncogenes, which are referred to as oncogene‐induced DNA damage foci (Mallette et al., 2007). Nuclear localization of γH2AX was detected in RET‐mice throughout the hair cycles (anagen, catagen, and telogen) in one‐month‐old and 20‐month‐old RET‐mice. In contrast, expression of γH2AX was not detected in WT‐mice at any hair cycle stage from 1 month and 20 months of age (Figure S6a). Oncogene‐induced DNA damage activates the p53‐p21 signaling pathway and/or the p16^ink4a^ signaling pathway, which has been shown to be critical for OIS (Lujambio, 2016). Expression of p16^ink4a^ was detected in KSCs in 20‐month‐old RET‐mice throughout the hair cycle stages (Figure S6b). In contrast, at one month of age, expression of p16^ink4a^ was detected at the anagen phase but not at the catagen or telogen phase. Expression of p16^ink4a^ was not detected in WT‐mice at any hair cycle stages regardless of age (Figure S6b). Expression of p‐p53 and p21 was not detected in RET‐mice or WT‐mice at any stage and at any age (Figure S6c,d). These data indicated that there is a replicative stress, a mechanism commonly associated with OIS, in KSCs from senior RET‐mice.

### 2.4 Age‐related decrease in expression levels of ETs in bulges from RET‐mice

We next examined how senescent KSCs are correlated with hair graying via a decreased number of MSCs. We hypothesized that hair graying with decreased MSCs and descendant melanocytes is caused by the dysfunctional interaction between KSCs and MSCs in RET‐mice. Interaction between KSCs and MSCs through endothelin/EdnrB signaling has been reported to play an important role in the maintenance of MSCs; however, the effects of this interaction in relation to aging have not been investigated. Fluorescence intensity of ETs in bulges from 20‐month‐old RET‐mice was less than 30% of that in bulges from littermate WT‐mice, whereas the intensities were comparable in 1‐month‐old WT‐mice and littermate RET‐mice (Figure 3f,g), suggesting that levels of ETs in bulges decreased with aging in RET‐mice. Moreover, there was a significant negative correlation (*ρ* = −0.86, *p* < 0.01) between fluorescence intensities of ETs and p16^ink4a^ in each cell in telogen bulges from RET‐mice (Figure 3h), indicating decreased expression levels of ETs in p16^ink4a^‐positive cells. There was also a significant correlation (*ρ* = 0.80, *p* < 0.01) between fluorescence intensities of ETs per bulge and the number of MSCs (Figure 3i).

### 2.5 Acceleration of hair graying in RET‐mice with heterozygously deleted Ednrb expression

**Figure 4:**
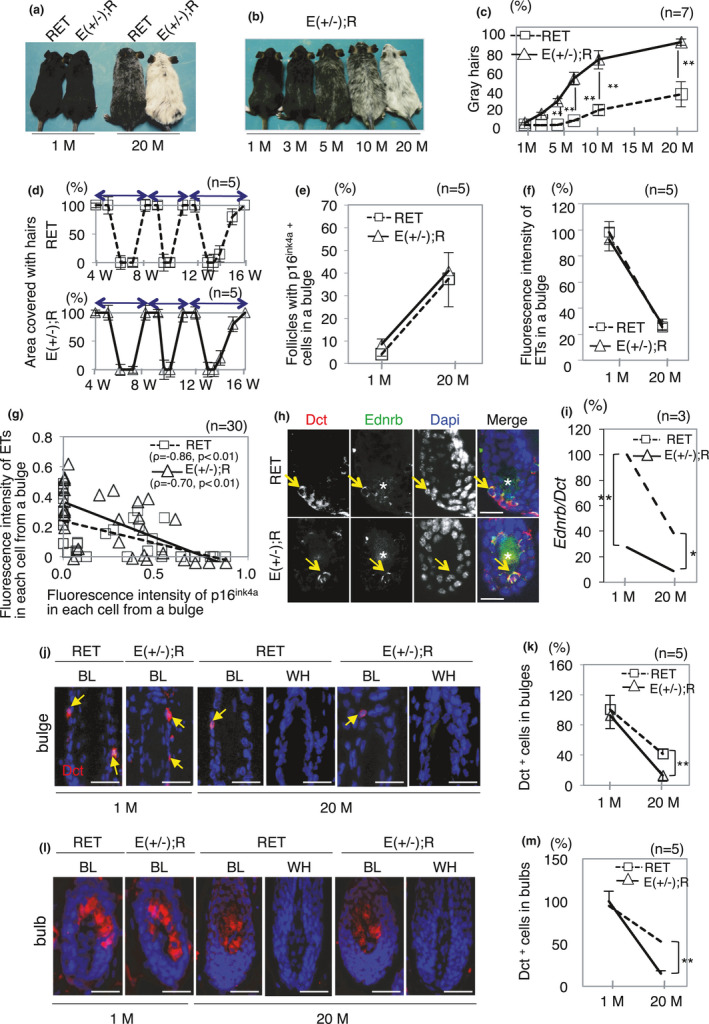
Accelerated hair graying in Ednrb(+/−); RET‐mice. (a) Representative macroscopic appearances of RET‐ and Ednrb(+/−); RET‐mice at 1 and 20 months of age. (b) Representative macroscopic appearances of Ednrb(+/−); RET‐mice at indicated ages. (c) Ratios of gray hairs (means ± SD, 100 hairs each) in RET‐mice (*n* = 7) and Ednrb(+/−); RET‐mice (*n* = 7) at indicated months of age. (d) Ratios (means ± SD) of the skin area covered with hairs after clipping in RET‐mice (top; *n* = 5) and Ednrb(+/−); RET‐mice (bottom; *n* = 5) from 4 weeks to 16 weeks of age. Telogen hairs were gently clipped at 7 weeks of age (1st clipping) in both model mice. Second and 3rd clippings were performed after hairs were fully regenerated at 10 and 14 weeks of age in both model mice. Two‐way arrows show one hair cycle. (e) Ratios (means ± SD) of follicles with p16^ink4a^‐positive cells in 50 telogen bulges each from RET‐mice (*n* = 5) and Ednrb(+/−); RET‐mice (*n* = 5) at 1 month of age and 20 months of age. (f) Ratios (means ± SD) of fluorescence intensity of ETs in telogen bulges (30 each) from RET‐mice at 20 months of age (*n* = 5) and Ednrb(+/−); RET‐mice at 1 month of age (*n* = 5) and 20 months of age (*n* = 5) to that in 30 telogen bulges from RET‐mice at 1 month of age (*n* = 5). (g) Correlations between fluorescence intensities of ETs and p16^ink4a^ monitored in the same cells of 30 individual cells in telogen bulges from RET‐mice and Ednrb(+/−); RET‐mice at 1 and 20 months of age. (h) Representative results for expression of Ednrb (green) and Dct (red) in telogen bulges from RET‐ and Ednrb(+/−); RET‐mice at 3 weeks of age. Yellow arrows indicate Ednrb and Dct double‐positive cells. (i) Ratios (means ± SD) of *Ednrb* expression levels normalized by *Dct* in telogen bulges (100 each) isolated by laser capture microdissection from RET‐mice at 20 months of age (*n* = 3) and Ednrb(+/−); RET‐mice at 1 month of age (*n* = 3) and 20 months of age (*n* = 3) to that from RET‐mice at 1 month of age (*n* = 3). (j) Representative results of Dct (red) expression in telogen bulges from black (BL) and white (WH) hair follicles of RET‐ and Ednrb(+/−); RET‐mice at 1 and 20 months of age. Arrows indicate Dct‐positive cells (MSCs) in a bulge. (k) Ratios (means ± SD) of the total number of Dct‐positive cells (MSCs) in bulges (70 each) from RET‐mice at 20 months of age (*n* = 5) and Ednrb(+/−); RET‐mice at 1 month of age (*n* = 5) and 20 months of age (*n* = 5) to that in bulges from RET‐mice at 1 month of age (*n* = 5) are presented. (l) Representative results of Dct (red) expression in bulbs in black (BL) and white (WH) anagen follicles from RET‐ and Ednrb(+/−); RET‐mice at 1 and 20 months of age. (m) Ratios (means ± SD) of Dct‐positive cells (MSCs) in bulbs (20 each) from RET‐mice at 20 months of age (*n* = 5) and Ednrb(+/−); RET‐mice at 1 month of age (*n* = 5) and 20 months of age (*n* = 5) to that in bulges from RET‐mice at 1 month of age (*n* = 5). Nuclei were stained with DAPI (blue). * and **Significantly different (*p < 0.05; **p < 0.01) by the Mann–Whitney *U* test. Bars, 10 μm. W, weeks; M, months

We next tried to clarify the correlation between age‐related decrease in the expression level of ETs in KSCs and decreased number of MSCs in RET‐mice. Immunohistochemical analysis of more than 30 telogen bulges indicated exclusive expression of Ednrb protein in Dct‐positive cells in WT‐mice and RET‐mice (Figure S7A). qPCR analysis further showed that *Ednrb* expression level in telogen bulges of 20‐month‐old RET‐mice was significantly lower of that in littermate WT‐mice (Figure S7B). Based on these results, we hypothesized that the interaction between ETs in KSCs and Ednrb in MSCs in bulges is a major pathway for age‐related hair graying in RET‐mice. If our hypothesis is correct, hair graying must be further accelerated in RET‐mice with decreased Ednrb levels in MSCs. To confirm this hypothesis, we developed homozygously [Ednrb(−/−); RET‐mice] and heterozygously [Ednrb(+/−); RET‐mice] Ednrb‐deleted RET‐mice. Since Ednrb(−/−); RET‐mice congenitally develop white hairs and die from Hirschsprung disease within one month, Ednrb(+/−); RET‐mice were used in this study. Hair graying in 20‐month‐old Ednrb(+/−); RET‐mice was further accelerated compared to that in RET‐mice of the same age (Figure 4a right), whereas coat colors in 1‐month‐old Ednrb(+/−); RET‐mice were comparable to those in littermate RET‐mice (Figure 4a left). Hair graying in Ednrb(+/−); RET‐mice progressively developed with aging (Figure 4b). The ratio of gray hairs in 20‐month‐old Ednrb(+/−); RET‐mice reached >85% on average, while that in littermate RET‐mice was 35.6% (Figure 4c). Meanwhile, the ratio of gray hairs (8.6%) in 20‐month‐old Ednrb(+/−)‐mice without carrying activated RET (RFP‐RET) was comparable to that in littermate WT‐mice (Figure S8). These results suggest that RET‐mice and Ednrb(+/−); RET‐mice could be models for mild and severe age‐related hair graying, respectively.

There was no difference between hair cycles in RET‐mice and littermate Ednrb(+/−); RET‐mice (Figure 4d). Ratios of follicles with p16^ink4a^‐positive KSCs (Figure 4e) and fluorescence intensities of ETs (Figure 4f) in telogen bulges were comparable in RET‐mice and littermate Ednrb(+/−); RET‐mice. Correlations between fluorescence intensities of ETs and p16^ink4a^ in each cell in telogen bulges were also comparable in RET‐mice (*ρ* = −0.86, *p* < 0.01) and littermate Ednrb(+/−); RET‐mice (*ρ* = 0.70, *p* < 0.01) (Figure 4g). These results indicate that characteristics of the hair cycle and KSC‐related factors (Figure 4d–g) were comparable in RET‐mice and Ednrb(+/−); RET‐mice.

After confirming that Ednrb protein was exclusively expressed in Dct‐positive cells in telogen bulges from Ednrb(+/−); RET‐mice in addition to RET‐mice (Figure 4h), we showed that the *Ednrb* transcript expression level in Dct‐positive cells in laser‐microdissected telogen bulges from 1‐month‐old Ednrb(+/−); RET‐mice was 27.5% of that in littermate RET‐mice (Figure 4i). While the *Ednrb* transcript expression level in Dct‐positive cells in telogen bulges from 20‐month‐old RET‐mice was reduced to 38.5% of that in 1‐month‐old RET‐mice, the level in 20‐month‐old Ednrb(+/−); RET‐mice was only 8.8% of that in 1‐month‐old RET‐mice (Figure 4i). In contrast, *Ednrb* transcript expression levels in telogen bulges from WT‐mice at 1 month and at 20 months of age were comparable (Figure S7b). The ratio of Dct‐positive cells in bulges (MSCs) from 20‐month‐old Ednrb(+/−); RET‐mice was significantly lower than that in 20‐month‐old RET‐mice, while the ratios were comparable at 1 month of age (Figure 4j, k). The ratio of Dct‐positive cells in bulbs from 20‐month‐old Ednrb(+/−); RET‐mice was also significantly lower than that in 20‐month‐old RET‐mice, while the ratios were comparable at 1 month of age (Figure 4l,m). These data suggested that reduction of Ednrb expression in relation to aging accelerated hair graying.

### 2.6 Age‐related decrease in expression levels of ET‐1 in bulges

**Figure 5:**
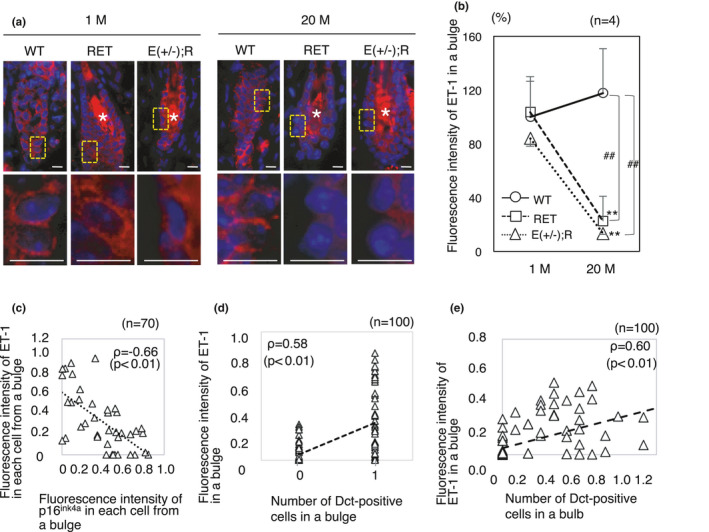
Decreased expression levels of ET‐1 in KSCs with aging in Ednrb(+/−); RET‐mice. (a) Representative results of ET‐1 (red) expression in telogen bulges from WT, RET, and Ednrb(+/−); RET‐mice at 1 and 20 months of age. Bottom panels show magnified images of the boxed areas in the top panels. Nuclei were stained with DAPI (blue). Asterisks indicate nonspecific signals in the hair shafts. Bars, 5 μm. (b) Ratios (means ± SD) of fluorescence intensity of ET‐1 in telogen bulges (20 each) from WT (*n* = 4), RET (*n* = 4) and Ednrb(+/−); RET‐mice (*n* = 4) at 1 and 20 months of age. ** and ^##^significantly different between 1 M and 20 M (**p < 0.01) and between WT and RET‐mice and WT and Ednrb(+/−); RET‐mice (^##^ *p* < 0.01) by the Tukey–Kramer test. (c) Correlations between fluorescence intensities of ET‐1 and p16^ink4a^ monitored in the same cells of 70 individual cells in telogen bulges from Ednrb(+/−); RET‐mice at 20 months of age. (d) Correlation between fluorescence intensity of ET‐1 and number of Dct‐positive cells detected in the same bulge region (100 follicles) from single tissue sections of 20‐month‐old Ednrb(+/−); RET‐mice. (e) Correlations between ratios of fluorescence intensities of ET‐1 in telogen bulges and number of Dct‐positive cells in bulbs from the same follicle (*n* = 100) in Ednrb(+/−); RET‐mice

We next tried to identify ETs that directly correlated with hair graying in Ednrb(+/−); RET‐mice. Our qPCR analysis showed that only *ET*‐*1* was down‐regulated in telogen bulges from 20‐month‐old Ednrb(+/−); RET‐mice, and its level was 70.7% lower than that in telogen bulges from 1‐month‐old Ednrb(+/−); RET‐mice (Figure S9). We further quantified the fluorescence intensity of ET‐1 protein in bulges from each mouse at 1 month and 20 months of age. There were no statistically significant differences between WT, RET and Ednrb(+/−); RET‐mice at 1 month of age. On the other hand, at 20 months of age, there was a significant decrease of ET‐1 fluorescence intensity in RET‐mice and Ednrb(+/−); RET‐mice compared to that in WT‐mice (Figure 5 a, b). There was a significant correlation between fluorescence intensity level of ET‐1 and p16^ink4a^ expression in each bulge cell from Ednrb(+/−); RET‐mice (*ρ* = −0.66, *p* < 0.01) (Figure 5c). There were also significant correlations of ET‐1 expression level with number of MSCs in a bulge (*ρ* = 0.58, *p* < 0.01) (Figure 5d) and number of descendant melanocytes in a bulb (*ρ* = 0.60, *p* < 0.01) (Figure 5e) from Ednrb(+/−); RET‐mice.

We next examined the expression of ET‐3 protein in our model mice since it is also known that ET‐3 stimulates hair pigmentation through Ednrb (Kurita et al., 2005). ET‐3 protein expression was confirmed in secondary hair germ at 3 days after depilation in WT‐mice as was previously reported (Figure S10a, Li et al., 2017). Notably, however, there was no ET‐3 expression in the bulge. We further confirmed that there was no ET‐3 protein expression in telogen bulges regardless of age (1 M and 20 M) from both WT‐mice and RET‐mice (Figure S10b). These data suggested that ET‐1 might be a main regulator of RET‐mediated hair graying in our model mice.

### 2.7 Mechanism for hair graying in humans

**Figure 6:**
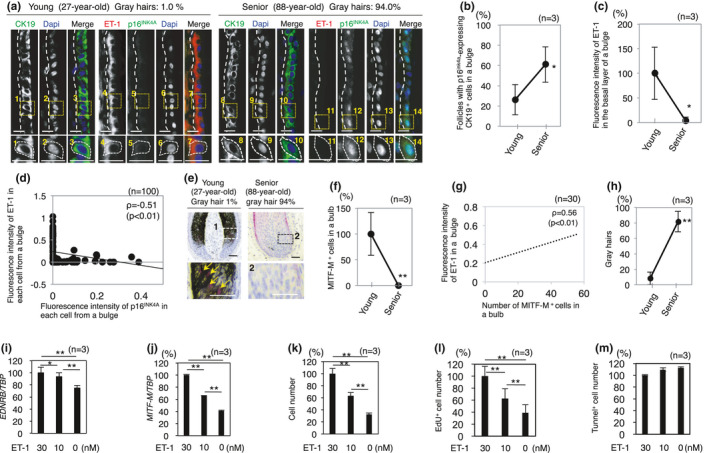
Mechanism for hair graying in humans. (a‐h) Results for human scalp samples (*n* = 10) from young people (Young, *n* = 3) at 27 years of age (a, e) and 27–40 years of age (b‐d, f‐h) with limited hair graying and from senior people (Senior, *n* = 7) at 88 years of age (a, e) and 61–90 years of age (b‐d, f‐h) with remarkable hair graying are presented. (a) Representative results of cytokeratin 19 (CK19) (green), p16^INK4A^ (green), and ET‐1 (red) expression in anagen bulges in the scalp. Nuclei were stained with DAPI (blue). p16^INK4A^ (green) and ET‐1 (red) were double‐stained, and serial sections were used for CK19. White broken lines in top panels show the border between the basal layer (right) and suprabasal layer (left). Bottom panels show magnified images of the boxed areas in the top panels. White dotted lines in the bottom panels show an outline of each cell in the basal layer. (b) Ratios (means ± SD) of follicles with p16^INK4A^ ‐positive KSCs (CK19‐positive cells) in anagen bulges from young people (80 follicles from 3 people) and senior people (46 follicles from 3 people). (c) Ratios (means ± SD) of fluorescence intensity of ET‐1 in KSCs in the basal layer of anagen bulges from senior people (24 follicles from 3 people) to that from young people (23 follicles from 3 people). (d) Correlation between fluorescence intensities of ET‐1 and p16^ink4a^ monitored in the same cell of 100 individual cells from CK19‐positive cells (KSCs) in anagen bulges. (e) Representative results of MITF‐M expression in anagen hair bulbs from young people (27 years old) and senior people (88 years old). Bottom panels (1, 2) show magnified images of the boxed areas in the upper panels. Arrows indicate MITF‐M‐positive cells (purple) in hair bulbs. (f) Ratios (means ± SD) of MITF‐M‐positive cells in hair bulbs (20 follicles each) from senior people (*n* = 3) to that in hair bulbs from young people (*n* = 3). (g) Correlation between fluorescence intensities of ET‐1 in anagen bulges and number of MITF‐positive cells in anagen hair bulbs from young and senior people. (h) Ratios of gray hairs (means ± SD, 100 hairs each) from young people (*n* = 3) and senior people (*n* = 3). (i–m) Ratios (means ± SD) of transcript expression levels of *ENDBR* (i) and *MITF*‐*M* (j), cell number (k), number of EdU‐positive cells (l), and number of Tunnel‐positive cells (m) in NHEMs (*n* = 3) in the presence (10 and 30 nM) or absence of ET‐1 for 72 h are presented. * and **Significantly different (*p < 0.05; **p < 0.01) by the Mann–Whitney *U* test (b, c, f, h) and Tukey–Kramer test (i–m). Bars, 10 μm (a) and 50 μm (e). Y, years

It has been reported that CK19‐positive cells in basal layers of bulges are KSCs in humans (Michel et al., 1996). Cells that are positive for the melanocyte‐specific isoform of the microphthalmia‐associated transcription factor (MITF‐M) in basal layers of bulges and bulbs are MSCs and descendant melanocytes, respectively, in humans (Nishimura et al., 2005). CK19‐positive cells (KSCs) in scalp hairs were detected in basal layers of bulges from young and senior people by our immunohistochemical analysis (1–3 and 8–10 in Figure 6a). Expression of p16^INK4A^ and ET‐1 in basal layers of bulges was also detected by our immunohistochemical analysis using serial sections from young and senior people (4–7 and 11–14 in Figure 6a). The ratio of follicles with p16^INK4A^‐expressing cells in basal layers of bulges from senior people was significantly higher than that in basal layers of bulges from young people (Figure 6b). Ratios of the fluorescence intensities of ET‐1 in basal layers of bulges from senior people were less than 4% of those in young people (Figure 6c). There was a significant correlation (*ρ* = −0.51, *p* < 0.01) between ET‐1 and p16^INK4A^ fluorescence intensities in cells in basal layers of bulges from young and senior people (Figure 6d). Since we could not detect MITF‐M‐positive cells in basal layers of bulges (MSCs) in the scalps of young and senior people, we further analyzed MITF‐M‐positive cells in bulbs (descendant melanocytes). Ratios of descendant melanocytes in senior people were less than 1% of those in young people (Figure 6e,f). There was a significant correlation (*ρ* = 0.56, *p* < 0.01) between fluorescence intensity of ET‐1 in a bulge and number of MITF‐M‐positive cells in bulbs (Figure 6g). The percentage of gray hairs in senior people was 9.8‐fold higher than that in young people (Figure 6h). To obtain more solid evidence for the interaction between ET‐1 and survival of melanocytes, we performed an *in vitro* study and found significant decreases in transcript expression levels of *EDNRB* and *MITF*‐*M*, which are melanocyte survival factors, in normal human melanocytes (NHEMs) in association with decreased ET‐1 concentration (Figure 6i,j). Moreover, the decreases in ET‐1 concentration caused a decrease in the number of NHEMs (Figure 6k). This decrease in cell number was due to the reduction of cell proliferation (decreased EdU incorporation, Figure 6l) and not by increased cell death (Figure 6m).

Taken together, our results obtained in humans suggest that decreased expression levels of ET‐1 in senescent KSCs in bulges promote hair graying via a decrease in the number of descendant melanocytes in hair bulbs.

## 3 DISCUSSION

We demonstrated hair graying derived from constitutively activated RET kinase‐mediated acceleration of hair cycles. RET‐mice showed involvement of OIS in senescence phenotype, γH2AX positive, suggesting replicative stress in KSCs. However, we did not observe activation of p53‐p21, which is one of the most important pathways in OIS (Figure S6). This resembles the reports showing that there is little p53 in benign or dysplastic nevi and that p53 abundance seemed to increase only in advanced melanoma (Mackenzie Ross et al. 2013; Gray‐Schopfer et al. 2006; Lassam et al. 1993; Stedanaki et al. 2008). We also noticed that the hair graying phenotype with an accelerated hair cycle may be associated with a darker back skin color (Figure 2b), which is reminiscent of the phenotype of transgenic mice ectopically expressing activated mutant NRas^Q61K^ in melanocyte lineages under the tyrosinase promoter–enhancer, resulting in hyperproliferation of melanocytes in the dermis (Ackermann et al., 2005; Li et al., 2012). The back skin of RET‐mice revealed melanocytes in the hair follicles, dermis, and fatty tissues, resembling the NRas^Q61K^ ‐mice phenotype (Figure S11).

Although previous studies suggested that increased levels of ETs in KSCs promote proliferation of MSCs via interaction between ETs and Ednrb in young genetically modified mice, there is very limited information about age‐related alteration of the interaction between ETs and Ednrb and its biological significance. We found significantly higher protein expression levels of ETs in those bulges with black hairs than in those with white hairs in RET‐mice (Figure S12). In addition to a more than 70% decrease in the expression of ETs in bulges, more than 60% and more than 90% decreases in the levels of Ednrb expression in MSCs were found in aged RET‐mice with mild hair graying and aged Ednrb(+/−); RET‐mice with severe hair graying, respectively. We also found strong correlations between fluorescence intensity of ETs and number of MSCs in bulges of RET‐mice (Figure 3i) and Ednrb(+/−); RET‐mice (Figure S13). Together with our results showing that hair graying in 20‐month‐old Ednrb(+/−)‐mice with intact expression of ETs in bulges and a 75.8% decrease in Ednrb expression in MSCs was comparable to hair graying in littermate WT‐mice (Figure S14), our results suggest that the combination of RET‐mediated decrease in expression of ETs in bulge KSCs and decrease of Ednrb expression in MSCs is involved in age‐related depletion of MSCs and age‐related hair graying in our model mice.

Previous studies showed decreased levels of various receptors following decreased levels of ligands (Miller et al., 2015). Previous studies also showed an interaction between ETs in KSCs and Ednrb in MSCs (Rabbani et al., 2011). Decreased ET‐1‐mediated decrease of Ednrb expression may be possible because a previous in vitro study showed that Ednrb expression level in melanocytic cells was decreased by reduction of ligand stimulation through a competitive inhibitor (BQ788) for ET‐1 (Lahav et al., 2004). Our study also showed that decreased ET‐1 resulted in decreases in the expression levels of EDNRB and MITF‐M as well as the number of proliferating melanocytes in cultured human melanocytes. Taken together, these results suggest a mechanism for hair graying through a decreased level of Ednrb expression (receptor) in MSCs caused by a decreased level of ET‐1 (ligand) in KSCs. In addition, no expression of Ret in MSCs throughout the hair cycle in RET‐mice (Figure S1) and comparable coat colors of Ednrb(−/−); RET‐mice and homozygously Ednrb‐deleted mice [Ednrb(−/−)‐mice] (Figure S15) suggest that compensation of Ednrb signaling by constitutively activated RET signaling is limited.

Hair graying with decreases of MSCs and descendant melanocytes in RET‐mice is in accordance with previously reported results for hair graying in humans, suggesting that RET‐mice could be a novel model mouse line for hair graying. Moreover, our results suggested a mechanism that promoted senescence of KSCs by increased hair cycles, decreased expression level of ET‐1 in senescent KSCs, decreased Ednrb expression level in melanocytes caused by decreased ET‐1 level, and decreased number of descendant melanocytes in hair bulbs, resulting in promotion of hair graying in our model mice (Figure S16). Increased regeneration of KSCs derived from cumulative hair cycles with aging has been observed in both mice and humans（Cotsarelis, 2006; Schneider et al., 2009). In the human scalp, it has been suggested that one hair cycle is about 2–6 years (Kligman, 1961). Ten hair cycles would be around 40 years of age on average, when people start to recognize hair graying (Keough & Walsh, 1965; Van Neste & Tobin, 2004). Ten hair cycles in our RET‐mice is about 10 months of ages, when we first observed obvious hair graying (Figure 1a). Therefore, the mechanism of hair graying in our model mice via an increase of cumulative hair cycles may be applicable for age‐related hair graying in humans, although further study is needed.

## 4 EXPERIMENTAL PROCEDURES

### 4.1 Mice

Ednrb(+/−)‐mice provided from the Jackson Laboratory and originally developed RET‐mice and Ednrb(+/−); RET‐mice (Kato et al., 1998; Kumasaka et al., 2010) were used in this study. This study was approved by the DNA Advisory Committee in Chubu University (approval no. 12‐06‐01) and Nagoya University (approval no. 16‐73), Japan. The study was also approved by the Animal Care and Use Committee in Chubu University (approval no. 2810030) and Nagoya University (approval no. 31233), Japan. Animal studies were not conducted blind but randomly assigned to all experiments.

### 4.2 Humans

Paraffin blocks with intact human scalp were obtained from the Department of Dermatology, Nagoya University Graduate School of Medicine. This study was approved by the Ethical Committee in Chubu University (approval no. 260019) and Nagoya University (approval no. 2013‐0070). Written informed consent was obtained from all participants.

### 4.3 Morphological analysis with a light microscope and a fluorescent microscope

Immunohistochemical analysis was performed according to the method previously described (Ohgami et al., 2010). Further information is provided in the section of Appendix S1.

### 4.4 Laser capture microdissection

Bulge areas in hair follicles were isolated from cryosections (7 µm) by laser capture microdissection (LCM) using a P.A.L.M. MBIV system (Carl Zeiss).

### 4.5 Quantitative polymerase chain reaction (qPCR)

qPCR with SYBR green was performed as previously reported (Ohgami et al., 2010). Details of the methods used for qPCR are provided in the expanded information.

### 4.6 Cell culture

Primary NHEMs (KURABO) were cultured in the presence or absence of ET‐1 (WAKO) according to the method previously shown (Yajima et al., 2017).

### 4.7 Statistical analysis

Statistical differences between two groups and among more than two groups were analyzed by the two‐sided Mann–Whitney *U* test and Steel‐Dwass test, respectively, because of no normal distribution. Analysis of variance by the *F* test showed no significant differences. Correlation was evaluated by Spearman's rank‐correlation coefficient. All statistical analyses were performed using JMP Pro (version 11.0.0; SAS Institute).

## CONFLICT OF INTEREST

This work was supported in part by a grant from Hoyu Co., Ltd. N.T. is an employee and shareholder of Hoyu Co., Ltd. The situation does not alter the authors’ adherence to the policies of the journal on sharing data and materials.

## AUTHOR CONTRIBUTIONS

M.Kato. developed two types of model mice for hair graying; M.I. and M.Kato. designed the study; M.I. performed all experiments and analysis of all data; I.Y., N.O., N. T., A.T., Y.G., M.Y.K., A.P‐B., M.Kono., M.A., and M.T. gave experimental support; M.I. A.T. and M.Kato wrote this paper.

## Supporting information

Fig S1‐S16

Appendix S1

## Data Availability

The data that support the findings of this study are available from the corresponding author upon reasonable request.
