# Supplementary material for: Hair graying with aging in mice carrying oncogenic RET
Source: Aging Cell. 2020 Nov 7;19(11):e13273. doi: 10.1111/acel.13273 (PMC7681064; doi:10.1111/acel.13273)
Supplement: Supplementary file 1 — Fig S1‐S16 [file ACEL-19-e13273-s001.pdf]

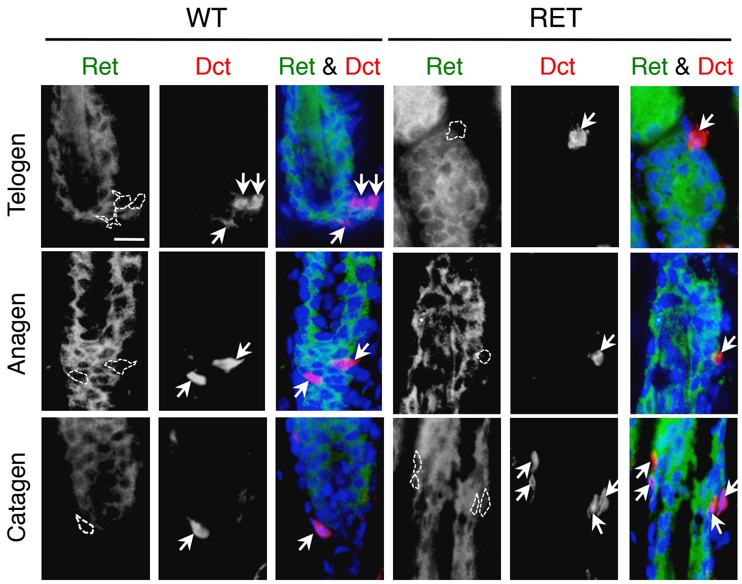

Supplemental Fig.1

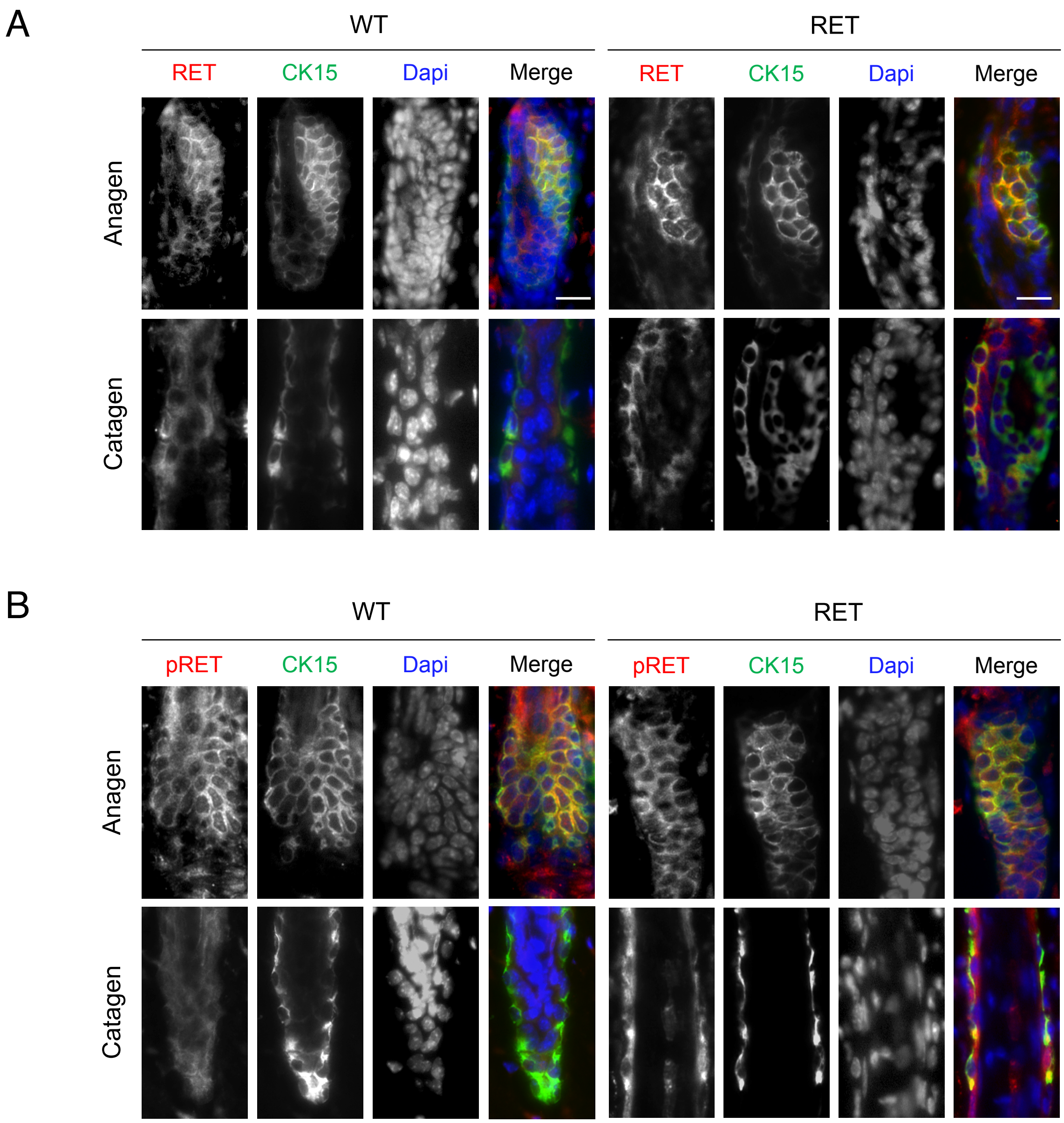

Supplemental Fig.2

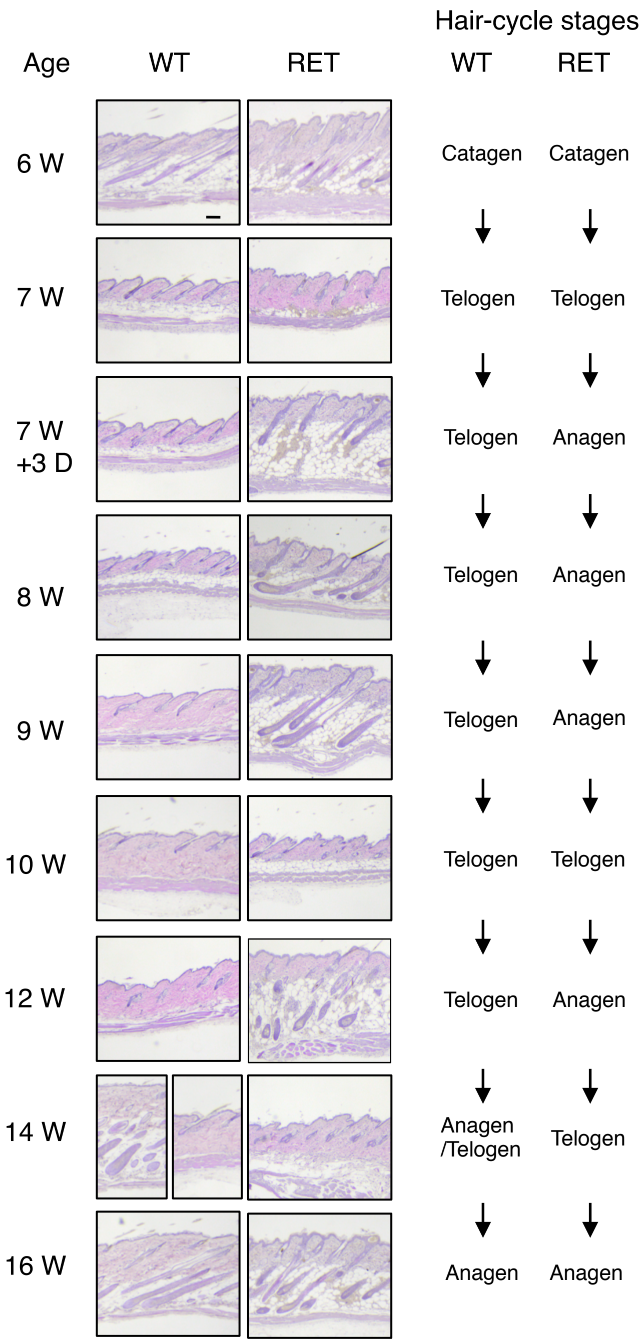

Supplemental Fig.3

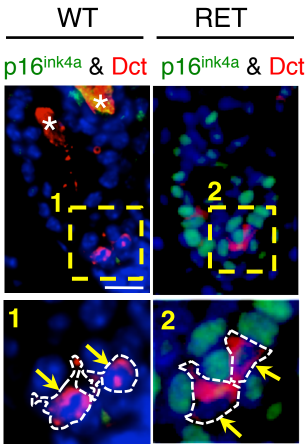

Supplemental Fig.4

A

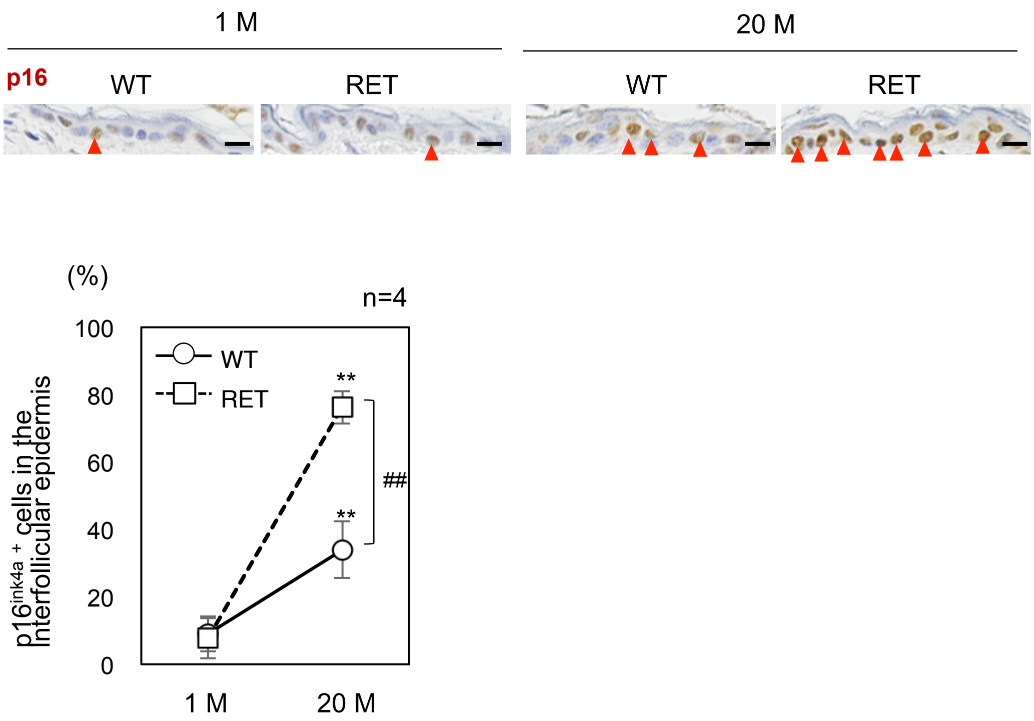

Supplemental Fig.5

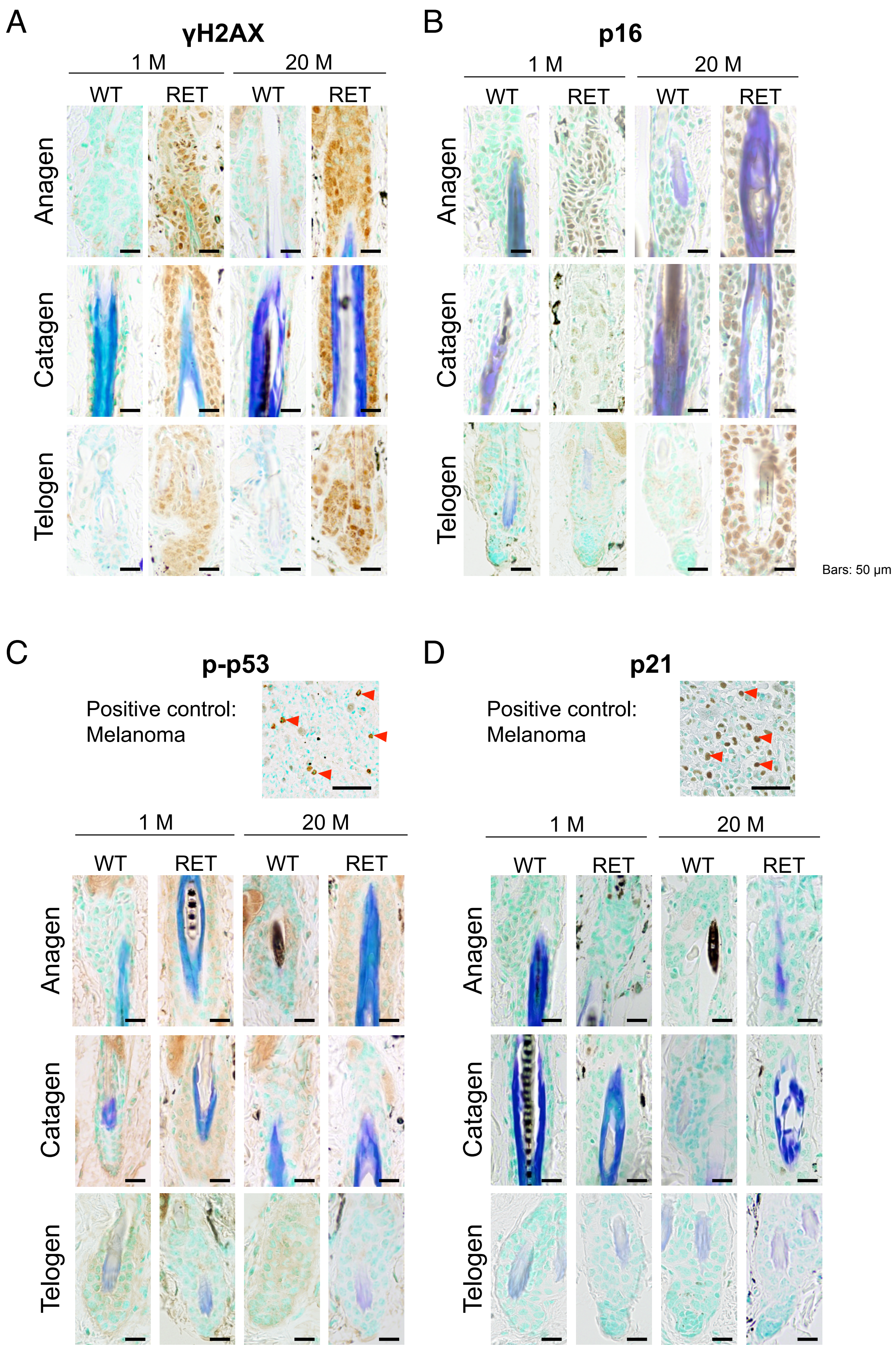

Supplemental Fig.6

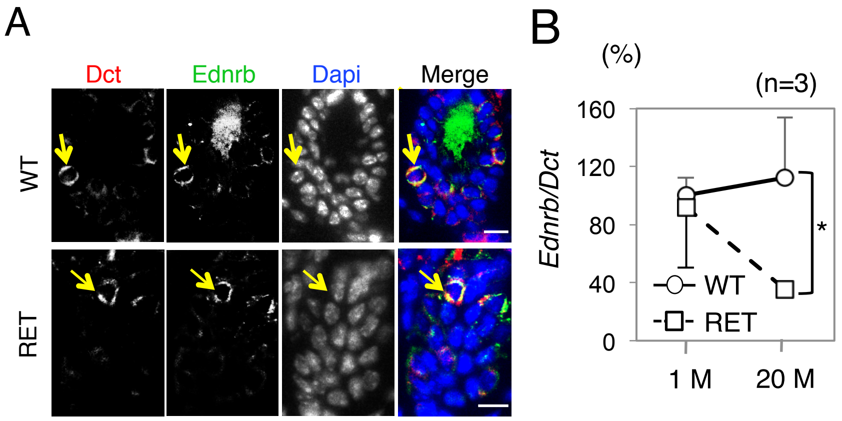

Supplemental Fig. 7

A

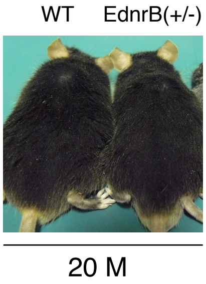

B

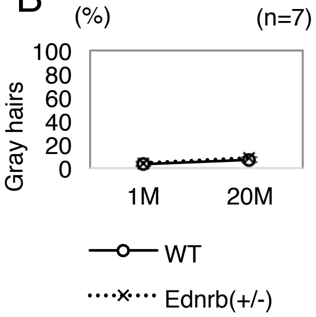

Supplemental Fig. 8

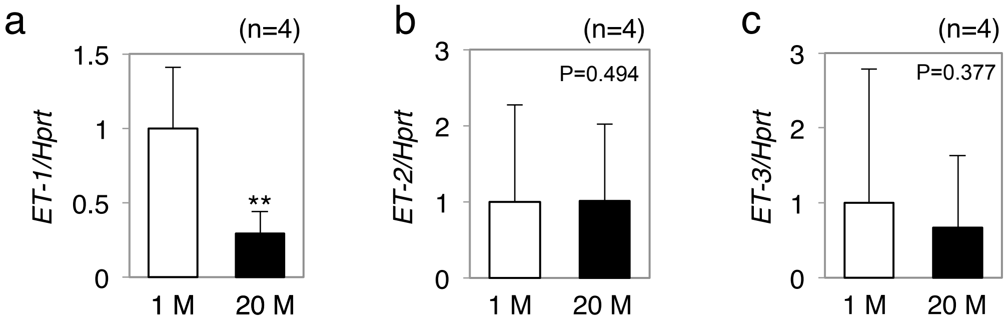

Supplemental Fig. 9

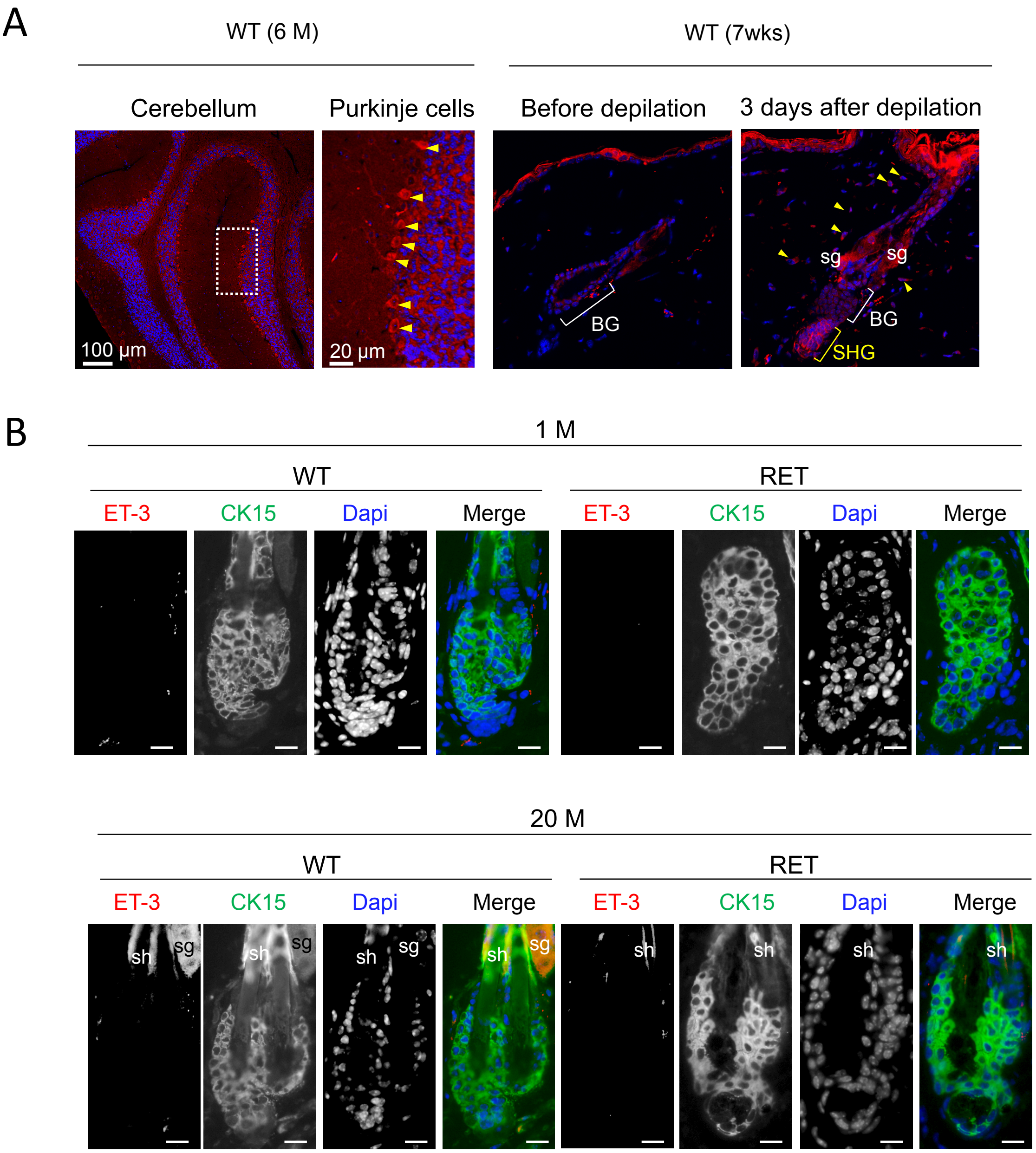

Supplemental Fig. 10

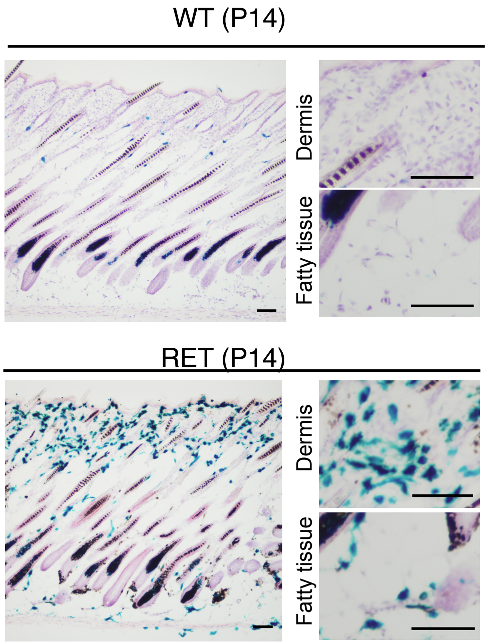

Melanocytes (LacZ) and melanin were distributed in dermis and fatty tissue as well as in hair follicles in RET-mice.

Supplemental Fig.11

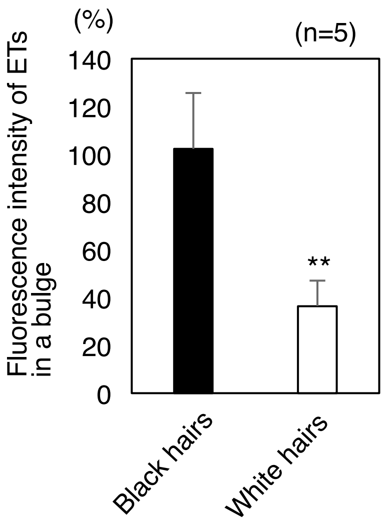

Supplemental Fig.12

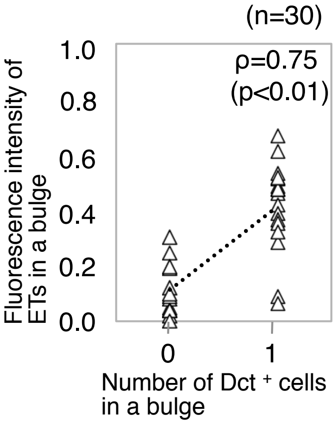

Supplemental Fig.13

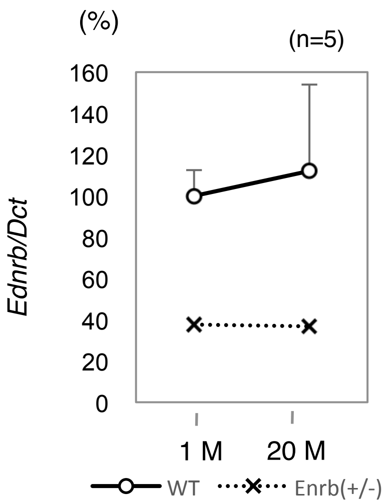

Supplemental Fig.14

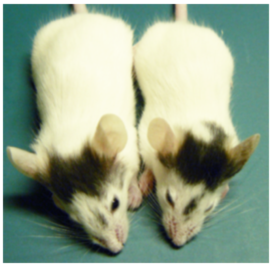

Ednrb(-/-)   Ednrb(-/-);RET

Supplemental Fig. 15

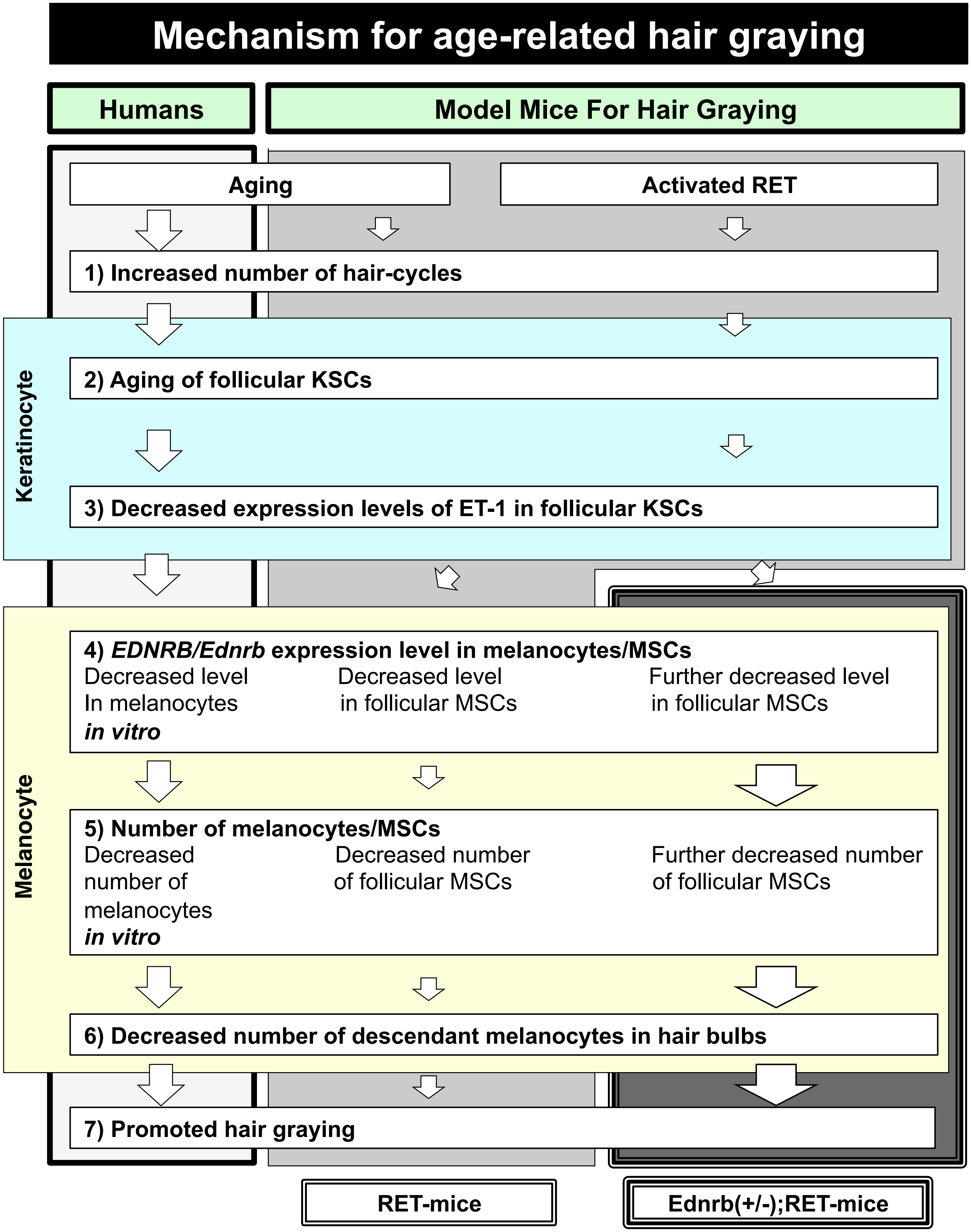

Supplemental Fig. 16
