# Supplementary material for: Hair graying with aging in mice carrying oncogenic RET
Source: Aging Cell. 2020 Nov 7;19(11):e13273. doi: 10.1111/acel.13273 (PMC7681064; doi:10.1111/acel.13273)
Supplement: Supplementary file 2 — Appendix S1 [file ACEL-19-e13273-s002.docx]

**Supplemental Experimental Procedures and legends for Supplemental Figures**

**Hair graying with aging in mice carrying oncogenic *RET***

Machiko Iida, Akira Tazaki, Ichiro Yajima, Nobutaka Ohgami, Nobuhiko Taguchi, Yuji Goto, Mayuko Y. Kumasaka, Armelle Prevost-Blondel, Michihiro Kono, Masashi Akiyama, Masahide Takahashi and Masashi Kato

**Supplemental Methods**

**Mice.** Dopachrome tautomerase (Dct)-lacZ transgenic mice were kindly provided by Dr. Ian J. Jackson (Mackenzie, Jordan, Budd, & Jackson, 1997).

**Dct-lacZ and SA-ß-gal staining.** Dct-lacZ staining was performed following the method previously described (Nishmura et al., 2002)^4^. SA-ß-gal staining was performed using cryosections according to the manufacturer’s protocol (Senescence Detection Kit, Medical & Biological Laboratories).

**Determination of gray hairs.** Hairs that were shown by microscopic analysis using WinROOF (Mitani Corporation) to have less than 40% of melanin content of fully pigmented hairs were defined as gray hairs in mice, according to the method previously described (Arck, P. C. et al., 2006).

**Immunohistochemistry.** Anti-RET (1:100, #18121, IBL), anti-phosphorylated tyrosine 905 (Y905) in RET protein (1:100, AF3269, R&D Systems), anti-phosphorylated Y1062 in RET protein (1:150, ab51103, Abcam), anti-CK15 (1:1000, PCK-153P, Covance), anti-p16^ink4a/INK4A^ (1:3000, F-12, Santa Cruz), anti-Dct (1:100, D-18, Santa Cruz), anti-endothelins 1/2/3 (1:100, Santa Cruz), anti-ET-1 (1:100, H-38, Santacruz), anti-Ednrb (1:100, LMC-57, MBL), anti-CK19 (1:200, RCK108, DAKO), anti-MITF-M (1:1000, MAB3747, Chemi-Con), Anti-γH2AX (Phospho S139) (1:200, ab81299, Abcam), anti- p53 (Phospho S15) (1:250, ab1431, Abcam) and anti-p21 (1:1000, ab188224, Abcam) were used as first antibodies for paraffin sections. Anti-RET antibodies were used according to our previous reports (Kato et al., 2001; Ohgami et al., 2010). For double staining of dct (1:500, D-18, Santacruz) and ET-1 (1:100, ab2786, Abcam), paraffin sections were treated with Blocking One Histo (Nakarai tesque) for antigen retrieval as previously reported (Ohgami et al., 2010)^3^. For double staining of Dct and Ednrb or p16^ink4a^ and single staining of MITF-M, paraffin sections were treated with 10 mM sodium citrate (pH 6.0) for antigen retrieval as previously reported (Ohgami et al., 2010). Envision kit/HRP detection system with diaminobenzidine (K1390, DAKO), Envision G|2 System/AP with Permanent Red (K535511, DAKO) or fluorescence-labeled secondary antibodies, Alexa Fluor 594 anti-goat IgG (A11058), Alexa Fluor 488 anti-rat IgG (A21208), Alexa Fluor 488 anti-chicken IgG (A11039), Alexa Fluor 488 (A21206) or 594 anti-rabbit IgG (A21207), and Alexa Fluor 488 (A21202) or 594 anti-mouse IgG (A21203) (1:1000, Invitrogen) were used for detection of target molecules. Counterstaining with hematoxylin or 4',6-diamidine-2'-phenylindole dihydrochloride (DAPI; 049-18801, WAKO) was performed. Fluorescence intensities of stained cells were evaluated using WinROOF (Mitani Corporation) by the method previously described (Ohgami et al., 2010). Immunohistochemical analysis was conducted blind. Randomization was conducted throughout the immunohistochemical analysis.

**Quantitative polymerase chain reaction (qPCR).** Total RNA isolation and cDNA synthesis from 100 follicular bulges per mouse dissected by laser capture microdissection were performed by using a NucleoSpin RNA XS kit (Macherey-Nagel) and SuperScript VILO (Invitrogen), respectively. Total RNA from NHEMs and HaCaT keratinocytes was isolated by using a High Pure RNA Purification Kit (Roche). cDNA was synthesized by using a PrimeScript RT reagent Kit (TaKaRa). The expression levels of mouse *ET-1*, *-2* and *-3* transcripts and human *P16^INK4a^*, *ET-1*, *EDNEB* and *MITF-M* transcripts were normalized by the expression levels of mouse *hypoxanthine phosphoribosyltransferase (Hprt)* and human *TATA-box-binding protein (TBP)*, respectively. The expression level of mouse *Ednrb* transcript was normalized by the transcript expression level of mouse *Dct.* Sequences of primers for mice were 5’-GCTTAATCCCTTTCAGAAAACAGCC-3’ and 5’-GGCAAGCAGAAGTAGAAACTGAAC-3’ for Ednrb, 5’-AATTCTTCAACCGGACATGC-3’ and 5’-GAACTTGCAGCCTCCACAAT-3’ for *Dct*, 5’-CTGCTGTTCGTGACTTTCCA-3’ and 5’-GACCCAGATGATGTCCAGGT-3’ for *ET-1*, 5’-CGTGACATCATCTGGGTGAA-3’ and 5’-CTTCGATGGCAGAAGGTAGC-3’ for *ET-*2, 5’-CAGGTCTGGGAAACAAGAGG-3’ and 5’-CTGGGAGCTTTCTGGAACTG-3’ for *ET-3*, and 5’-TATGTCCCCCGTTGACTGAT-3’ and 5’-CTTTGCTGACCTGCTGGATT-3’ for Hprt. Sequences of primers for humans were 5’-GCTCGTCCCTGATGGATAAA-3’ and 5’-CTGTTGCCTTTGTGGGAAGT-3’ for *ET-1*, 5’-GCCAGCGTCCTCATCTAT-3’ and 5’-GCCGTAAGGAGCTGTCTGTTC-3’ for *ET-2,* 5’-ATTGCCACCTGGACATCATT-3’ and 5’-GCAGGCCTTGTCATATCTCC-3’ for *ET-3,* 5’-ATCTGCGAATCTGCTTGCTT-3’ and 5’- TCCCGTCTCTGCTTTAGGTG-3’ for *EDNRB*, 5’-CCGTCTCTCACTGGATTGGT-3’ and 5’- TGGGCTTGCTGTATGTGGTA-3’ for *MITF-M*, and 5’-CACGAACCACGGCACTGATT-3’ and 5’- TTTTCTTGCTGCCAGTCTGGAC-3’ for *TBP*.

**References for Supplemental Methods**

Mackenzie, M. A., Jordan, S. A., Budd, P. S. & Jackson, I. J. Activation of the receptor tyrosine kinase Kit is required for the proliferation of melanoblasts in the mouse embryo. *Dev. Biol.* **192,** 99–107 (1997).

Ohgami, N. *et al.* c-Ret-mediated hearing loss in mice with Hirschsprung disease. *Proc. Natl. Acad. Sci. U. S. A.* **107,** 13051–13056 (2010).

Kato, M. Takeda K, Kawamoto Y, Tsuzuki T, Dai Y, Nakayama S, ... Nakashima I. RET tyrosine kinase enhances hair growth in association with promotion of melanogenesis. *Oncogene* **20** (51)**,** 7536–7541 (2001).

Arck, P. C. *et al.* Towards a “free radical theory of graying”: melanocyte apoptosis in the aging human hair follicle is an indicator of oxidative stress induced tissue damage. *FASEB J.* **20,** 1567–1569 (2006).

**Legends for Supplemental Figures**

**Supplemental Fig 1. Expression of c-Ret/RET in MSCs.** Expression of c-Ret/RET (Ret; green) and Dct (red)-positive cells in bulges (MSCs) at the telogen phase at 3 weeks of age, anagen phase at 4 weeks of age and catagen phase at 6 weeks of age. White broken lines show c-Ret/RET-negative cells. White arrows show Dct-positive cells. Bars, 10 μm.

**Supplemental Fig 2. Expression of c-Ret/RET in KSCs.** Representative results for expression of **(A)** c-Ret/RET (RET; red), cytokeratin 15 (CK15; green) and **(B)** c-Ret/RET protein phosphorylated at tyrosine 905 (pRet; red) in anagen and catagen bulges at 3 weeks of age are presented. Yellow merged with red and green indicates double staining of CK15 and c-Ret/RET or phosphorylated c-Ret/RET proteins in KSCs. Bars, 10 μm.

**Supplemental Fig 3. Microscopic appearances of hair cycles in WT-mice and RET-mice.** Microscopic results for hair cycles of WT-mice and RET-mice are presented. Histologically diagnosed stages of hair cycles in WT-mice and RET-mice from 6 weeks to 16 weeks of age are shown. A mixture of telogen and anagen follicles was obtained from WT-mice at 14 weeks of age. Bars, 100 μm. W, weeks; D, days.

**Supplemental Fig 4. Expression of p16^ink4a^ in MSCs.** Expression of p16^ink4a^ (green) and Dct (red) in telogen bulges (MSCs) from WT-mice and RET-mice at 10 months of age. Bottom panels **(1, 2)** show magnified images of the boxed areas presented by yellow broken lines in the top panels. White dotted lines and yellow arrows in the bottom panels show p16^ink4a^-negative and Dct-positive cells, respectively. Nuclei were stained with DAPI (blue). Bars, 10 μm.

**Supplemental Fig 5. Expression of p16^ink4a^ in interfollicular basal epidermal cells. (A)** Representative results for expression of p16^ink4a^ (red arrowheads) in the interfollicular basal epidermal cells of back skin from WT- and RET-mice at 1 month (1 M) and 20 months (20 M) of age. Nuclei were stained with methyl green. **(B)** Ratios (means ± SD) of p16^ink4a^-positive cells in the interfollicluar epidermis. 300 cells in the basal layer of interfollicular epidermis were analyzed in WT-mice (n=4) and RET-mice (n=4) at 1 month and 20 months of age. ** and ^##^, Significantly different between 1 month and 20 months of age (^**^p<0.01) and between WT-mice and RET-mice age (^##^p<0.01) by the Tukey-Kramer test. Bars, 10 μm. M, month.

**Supplemental Fig 6. OIS marker expression in bulges from in WT and RET-mice. (A-D)** Expression of γH2AX **(A)**, p16^ink4a^ **(B)**, phospholilated p53 **(C)**, p21**(D)** in anagen. catagen and telogen bulges from WT-mice and RET-mice at 1 month of age (1 M) and 20 months of age (20 M) are presented. Melanomas developed in mice were used as a positive control tissue for p-p53 and p21. Red arrowheads indicate p-p53/p21-positive nuclei. Nuclei were stained with methyl green. Bars, 50 μm.

**Supplemental Fig 7. Decreased expression levels of Ednrb in bulges from RET-mice with aging. (A)** Representative protein expression of Ednrb (green) and Dct (red) in telogen bulges from WT-mice and RET-mice at 3 weeks of age. Yellow merged with green and red indicates double-positive staining of Ednrb and Dct (Arrows). **(B)** Ratios (means ± SD) of *Ednrb* transcript expression levels divided by *Dct* transcript expression levels in telogen bulges (100 each) isolated by laser capture microdissection from WT-mice at 20 months of age (n=3) and RET-mice at 1 month of age (n=3) and 20 months of age (n=3) to that in telogen bulges from WT-mice at 1 month of age (n=3). Nuclei were stained with DAPI (blue). **, Significantly different (**p<0.01) by the Mann-Whitney *U* test. Bars, 10 μm. M, months.

**Supplemental Fig 8. Macroscopic appearance of gray hairs in EdnrB(+/-)-mice. (A)** Representative macroscopic appearances of WT and EdnrB(+/-)-mice at 20 months of age. **(B)** Ratios (means ± SD) of gray hairs (100 hairs were counted for each mice) in WT-mice (n=7) and EdnrB(+/-)-mice (n=7) at 1 and 20 months of age.

**Supplemental Fig 9. Expression of ETs in KSCs with aging in Ednrb(+/-);RET-mice. (A-C)** Ratios (means ± SD) of *ET-1* **(A)**, *ET-2* **(B)** and *ET-3* **(C)** transcript expression levels normalized by *Hprt* in telogen bulges (100 each) isolated by laser capture microdissection from Ednrb(+/-);RET-mice at 20 months of age (n=4) to those in at 1 month of age (n=4). Significantly different (**p<0.01) by the Mann-Whitney *U* test.

**Supplemental Fig. 10. Limited ET-3 protein expression in hair follicles. (A)** ET-3 protein expression in cerebellum (left) and in hair follicles 3days after depilation (right). ET-3 is expressed in purkinje cells (left, yellow arrowheads) and in dermis and secondary hair germ (SHG) but not in bulge (BG) in hair follicles (right, yellow arrowheads). **(B)** Limited ET-3 expression in KSCs in telogen bulge from 1 and 20 month-old WT and RET-mice. sh: hair shaft, sg: sebaceous gland. Bars, 20 µm.

**Supplemental Fig. 11. Melanocyte distribution in RET-mice.** Melanocytes (LacZ) and melanin were distributed in dermis and fatty tissues as well as in hair follicles in RET-mice. Bars, 100 µm.

**Supplemental Fig 12. Expression of ETs in black and white hairs of RET-mice.** Ratios (means ± SD) of fluorescent intensity of ETs in bulges (30 each) of black hairs and white hairs from RET-mice are presented. **, Significantly different (**p<0.01) by the Mann-Whitney *U* test.

**Supplemental Fig. 13. Correlation between ETs level and melanocyte number in Ednrb(+/-);RET-mice.** Correlation between fluorescence intensity of ETs and number of Dct-positive cells detected in the same bulge region (30 follicles) from single tissue section of 20 month old Ednrb(+/-);RET-mice.

**Supplemental Fig 14. Expression of Ednrb in MSCs of Ednrb(+/-)-mice.** Ratios (means ± SD) of *Ednrb* transcript expression levels normalized by *Dct* in telogen bulges (60 each) isolated by laser capture microdissection from Ednrb(+/-)-mice at 20 months of age (n=5) to those in at 1 month of age (n=5).

**Supplemental Fig. 15. Coat color of Ednrb(-/-) and Ednrb(-/-); RET-mice.** Representative coat color of Ednrb(-/-)-mouse (left) and Ednrb(-/-);RET-mouse (right) with genetic background of C57BL/6 mouse at three weeks of age are presented. Coat color was comparable between Ednrb(-/-)-mouse (left) and Ednrb(-/-);RET-mice (right).

**Supplemental Fig. 16. Hypothetical mechanisms for hair graying.** Hypothetical mechanisms for hair graying progressively developing with aging (age-related hair graying) are presented.
